# Field expedient stool collection methods for gut microbiome analysis in deployed military environments

**DOI:** 10.1128/msphere.00818-24

**Published:** 2025-05-15

**Authors:** Car Reen Kok, James B. Thissen, Michele Cerroni, David R. Tribble, Anthony Cancio, Sophia Tran, Christina Schofield, Rhonda E. Colombo, Tom Troth, Christie Joya, Tahaniyat Lalani, Nicholas A. Be

**Affiliations:** 1 Lawrence Livermore National Laboratory 4578, Livermore, California, USA; 2 Infectious Disease Clinical Research Program, Department of Preventive Medicine and Biostatistics, Uniformed Services University of the Health Sciences 1685, , Bethesda, Maryland, USA; 3 Navy Medicine Readiness and Training Command 680745 , Portsmouth, Virginia, USA; 4 The Henry M Jackson Foundation for the Advancement of Military Medicine, Inc., Bethesda, Maryland, USA; 5 Tripler Army Medical Center 8015, Honolulu, Hawaii, USA; 6 Madigan Army Medical Center 19933, Tacoma, Washington, USA; 7 United Kingdom Ministry of Defence 1755, London, England, United Kingdom; 8 University of Birmingham, Birmingham, United Kingdom; University of Michigan Medical School, Ann Arbor, Michigan, USA

**Keywords:** microbiome, metagenomics, sample collection, field study

## Abstract

Field expedient devices and protocols for the collection, storage, and shipment of stool samples in deployed settings are needed for the advancement of microbiome research in military health. Relevant assessments include the evaluation of microbiome signatures associated with susceptibility to travelers’ diarrhea and recovery of gut function following infection. However, inherent biases in microbial measurements due to preservatives and sampling methods are unclear and should be assessed for an accurate evaluation of the microbiome. We performed shotgun metagenomic sequencing and compared the microbiome composition in paired fecal samples collected using Flinters Technology Associates (FTA) cards and OMNIgene (OG) Gut tubes, prior to and during international travel, from 49 adult participants, 39 of whom remained asymptomatic and 10 experienced travelers’ diarrhea. Higher concentrations of nucleic acid and sequencing libraries were observed in OG samples. A majority of genera (82.9%) were detected with both methods, and detections of genera limited to one collection method were not highly prevalent across samples and were present in extremely low relative abundances (<0.01%). Differences in beta diversity were largely explained by inter-individuality of microbiome composition, followed by the effect of collection method and timepoint-disease states. Differential abundance analysis indicated that *Corynebacterium* and *Blautia* were consistently higher in abundance across all groups with FTA and OG collection, respectively. The observed differences in microbiome composition between methods suggest the need for consistent and standardized protocols within a study. Overall, the data presented here could help guide the future design of fecal microbiome study protocols in field and military deployment settings.

**IMPORTANCE:**

The assessment of field-deployable methods for fecal sample collection and storage is required to reliably capture samples collected in remote and austere locations. This study describes a comparative metagenomics analysis between samples collected by two different commercially available methods in a military-deployed setting. The results presented here are foundational for the future design of fecal microbiome study protocols in an operational context.

## INTRODUCTION

The advancement of microbiome research in military health has led to interest in the discovery of microbiome-derived biomarkers and perturbation recovery strategies to optimize the health, resilience, and performance of the warfighter (1–3). This includes studying the effects of military-relevant diseases, physical and emotional stressors, therapeutics, and diet on the gut microbiome of deployed individuals (4–7). Fecal samples are typically used as a non-invasive proxy of the gut microbiome, whereby multi-omics approaches, such as metagenomic sequencing and metabolomics, are applied to study microbial communities. Nonetheless, aside from biological differences, microbiome profiles are influenced by other factors, such as fecal collection, storage methods, and nucleic acid extraction protocols.

Immediate freezing after collection has been regarded as the gold standard for prolonged fecal preservation prior to nucleic acid extraction (8). However, this is not always feasible for studies that are conducted in remote locations where access to cold-chain storage is limited. The military environment is distinct, with operational constraints on top of hostile and remote conditions that would likely influence sample collection protocols. These challenges include limited infrastructure, unpredictable conditions, extreme temperatures, and atypical environmental contaminants. Furthermore, collection strategies that limit discomfort in austere settings are also important for study compliance.

Multiple studies have evaluated the taxonomic stability of the stool microbiome in a laboratory setting by comparing frozen stool to samples stored with different preservation methods at room temperature for days to months (9–14). Specifically, Flinters Technology Associates (FTA) cards and OMNIgene (OG) kits (DNA Genotek Inc.) have been evaluated as stable and convenient alternatives for ambient fecal storage with reported high concordance to immediate freezing (8, 11, 13, 15). One study in particular compared the efficacy of five different storage methods and found that 95% ethanol, FTA cards, and OG kits were effective for room temperature preservation for up to 8 weeks (8). However, alcohol-based methods, such as 95% ethanol, are susceptible to changes under temperature fluctuations encountered in the field. FTA cards and OG kits, on the other hand, are non-volatile and are likely suitable candidates for use in deployed environments. FTA consists of cellulose-based matrix paper that contains chemicals that lyse cells, denature proteins, and stabilize nucleic acid, while OG kits utilize a chelating agent-based solution for nucleic acid stabilization. Additionally, FTA cards have previously been shown to be effective for the detection and quantification of specific diarrheal pathogens from active-duty personnel, comparable to that of frozen stool (16–18).

In this study, we systematically compared and identified taxonomic distinctions between paired fecal metagenomes collected in operational field conditions using two commercially available and well-studied methods, FTA cards and OG kits. A blinded interim analysis was conducted on samples and data that were collected as part of a clinical trial evaluating the efficacy of a food supplement to maintain gut health during deployment or travel. While previous studies have evaluated the efficacy of different fecal preservation methods in a laboratory setting, similar assessments of fecal sample preservation in austere deployment and travel settings have not been performed. Given that the gut microbiome varies according to different populations and environmental exposures (5, 19–21), such data would be informative in identifying suitable sample storage methods for reliable and reproducible data in an operational context.

## RESULTS

### Military traveler’s diarrhea cohort and sample sequencing metrics

**Fig 1:**
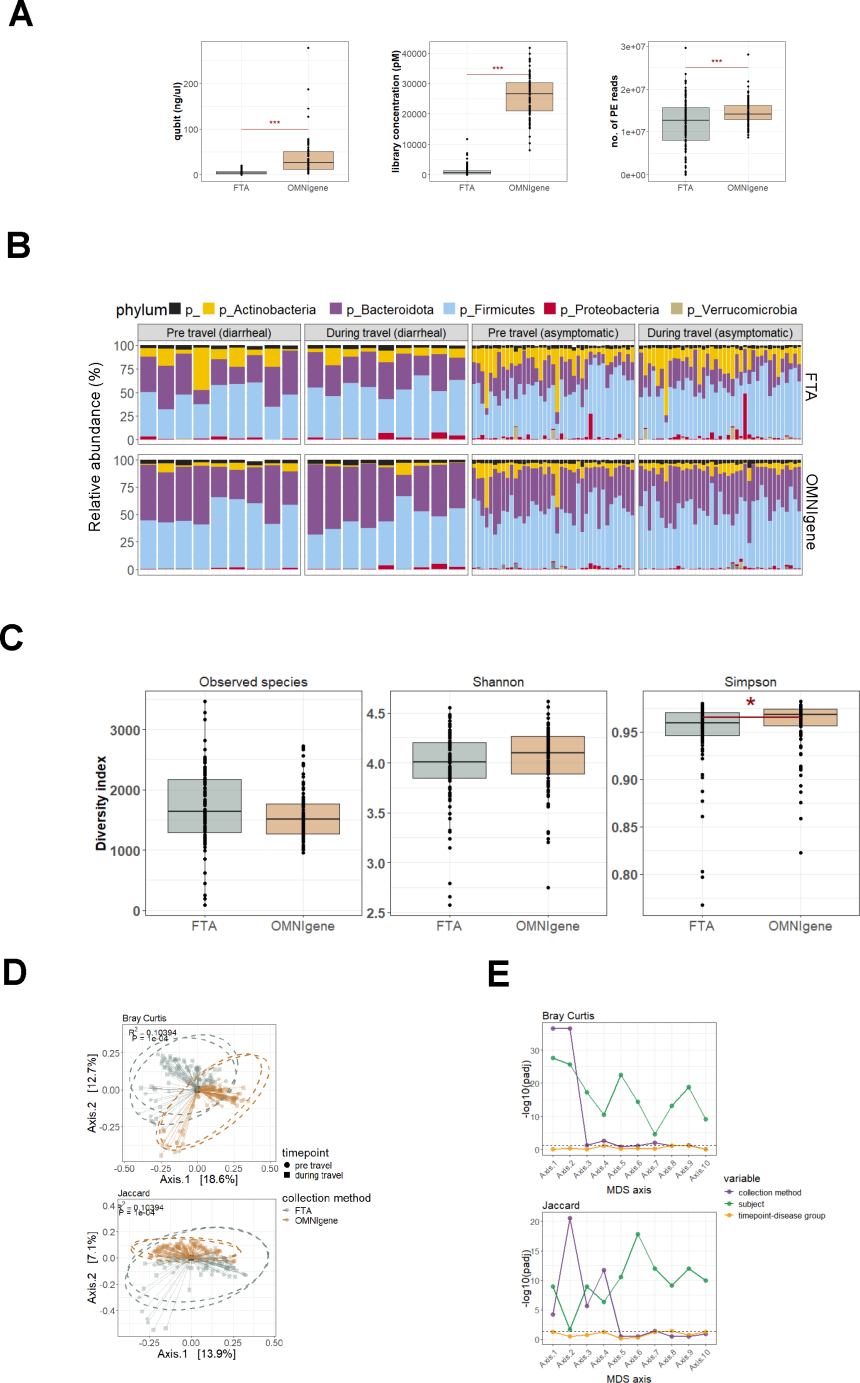
Comparative analyses of nucleic acid yield, sequencing metrics, and microbiome diversity between FTA and OG samples. (**A**) DNA concentration, sequencing library concentration, and the number of generated paired-end reads between FTA and OG (***P < 0.001). (**B**) Relative abundance of phyla detected across all FTA and OG samples. (**C**) Alpha diversity of samples measured using the number of observed species, Shannon index, and Simpson index (*P < 0.05). **D**) Beta diversity of samples measured using Bray-Curtis and binary Jaccard distances. PERMANOVA was used to determine significant differences between collection methods. (**E**) The effect of collection method, subject, and timepoint-disease group on the axes of the principal coordinate analysis of Bray-Curtis and binary Jaccard distances was estimated using an ANOVA of mixed linear models.

Forty-nine study participants were included in the analysis (Table 1), 39 of whom were asymptomatic during travel and 10 of whomexperienced travelers’ diarrhea. One individual who experienced traveler’s diarrhea was removed from downstream analyses due to a low number of sequencing reads (below 1,000 reads) in a pre-travel FTA sample post-quality filtering. All participants submitted paired pre-travel (i.e., FTA cards and OG kits) and during travel samples. The number of days between collection and nucleic acid extraction ranged from 6 to 81 days. Higher nucleic acid and sequencing library concentrations, along with a higher number of post-processed sequenced reads, were observed in OG compared to FTA samples (Fig. 1A). Both OG and FTA blank controls resulted in less than 20 post-processed reads, confirming the absence of background species that could confound downstream analyses.

**TABLE 1:** Cohort characteristics and sample storage conditions

Parameter	Total (*n* = 49)	Asymptomatic (*n* = 39)	Gut health disruption (*n* = 10)
Median age (IQR)	33 (26–39)	33 (26–39)	32 (26–40)
Male gender (%)	43 (87.8)	34 (87.2)	9 (90.0)
Race/ethnicity			
U.S.: Caucasian	23 (60.5)	17 (60.7)	6 (60.0)
U.S.: African American	7 (18.4)	7 (25.0)	0
U.S.: Asian	5 (13.2)	1 (3.6)	4 (40.0)
UK: Caucasian	11 (100)	11 (100)	0
Pre-travel FTA			
Duration of storage at room temperature (median day – IQR)	7 (5–30)	7 (5–55)	7.5 (6–19)
Duration between collection and extraction (median day – IQR)	17 (13–37)	21 (13–58)	15.5 (13–27)
Pre-travel OMNIgene			
Duration of storage at room temperature (median day – IQR)	7 (5–30)	7 (5–55)	7.5 (6–19)
Duration between collection and extraction (median day – IQR)	18 (14–37)	19 (14–57)	16.5 (13–27)
During travel FTA			
Duration of storage at room temperature (median day – IQR)	37 (20–55)	40 (21–60)	33 (9–45)
Duration between collection and extraction (median day – IQR)	53 (29–60)	54 (30–63)	47.5 (23–53)
During travel OMNIgene			
Duration of storage at room temperature (median days – IQR)	35 (19–55)	40 (21–60)	25.5 (9–42)
Duration between collection and extraction (median days – IQR)	52 (29–60)	56 (30–62)	37.5 (23–51)

### Differences in microbial profiles captured by FTA and OMNIgene

The five major gut phyla, Actinobacteria, Bacteroidota, Firmicutes, Proteobacteria, and Verrucomicrobia, were observed across all FTA and OG samples (Fig. 1B). At the phyla level, relative abundances of Proteobacteria (FTA: 3.07%, OG: 0.96%) and Actinobacteria (FTA: 14.95%, OG: 6.17%) were on average higher in FTA compared to OG samples, while Bacteroidetes was higher in OG samples (37.02%) compared to FTA (26.34%). Samples were grouped according to timepoint (pre-/during-travel) and disease state (travelers’ diarrhea/asymptomatic), and differences in microbial profiles between the two collection methods were evaluated by comparing microbial diversity and identifying differentially abundant species across groups.

Alpha diversity was measured through the number of observed species, Shannon index, and Simpson index. Simpson diversity was significantly higher in OG compared to FTA samples, while there were no differences in the number of observed species and Shannon diversity between collection methods (Fig. 1C). When comparing alpha diversity between collection methods per timepoint-disease state group, the only significant difference observed was in the Simpson diversity of FTA and OG pre-travel samples from subjects who remained asymptomatic, whereby OG samples were significantly higher (Fig. S1).

Beta diversity was evaluated using Bray-Curtis and binary Jaccard distances, and the first two axes were visualized using a principal coordinates analysis (PCoA) (Fig. 1D). Significant differences in collection method, subject, and timepoint/disease group were observed using a permutational analysis of variance (PERMANOVA) analysis on each beta diversity metric (*P* < 0.05) (Table 2). Inter-individuality explained the highest percentage of variability, followed by collection method and timepoint-disease group. Additionally, the significant effects of each variable on the first 10 PCoA axes were evaluated using an ANOVA of mixed linear models (Fig. 1E). Similar to the PERMANOVA results, subject/participant was observed to have significant impacts across all axes for both Bray-Curtis and binary Jaccard distances, followed by collection method. Timepoint-disease group did not have significant effects across all axes. To assess the efficacy of FTA and OG in preserving microbiome samples upon collection, Pearson correlation was calculated between the elapsed time from sample collection to DNA extraction and the Bray-Curtis dissimilarity index for paired FTA and OG samples (Fig. S2). A correlation coefficient of 0.085 (*P* = 0.41) was obtained, indicating no significant linear relationship. This suggests that time does not impact microbial community differences between FTA and OG samples and that both methods are effective in maintaining sample stability over the observed period.

**TABLE 2:** Estimated percentage of variability explained (R2) by collection method, subject and timepoint-disease group using Bray-Curtis and Jaccard binary distance metrics

Variable	Bray-Curtis		Jaccard	
	R^2^	Pr(>F)	R^2^	Pr(>F)
Collection method	0.10394	<0.0001	0.04444	<0.0001
Subject	0.58494	<0.0001	0.44302	<0.0001
Timepoint-disease group	0.00672	0.032	0.01232	0.002

**Fig 2:**
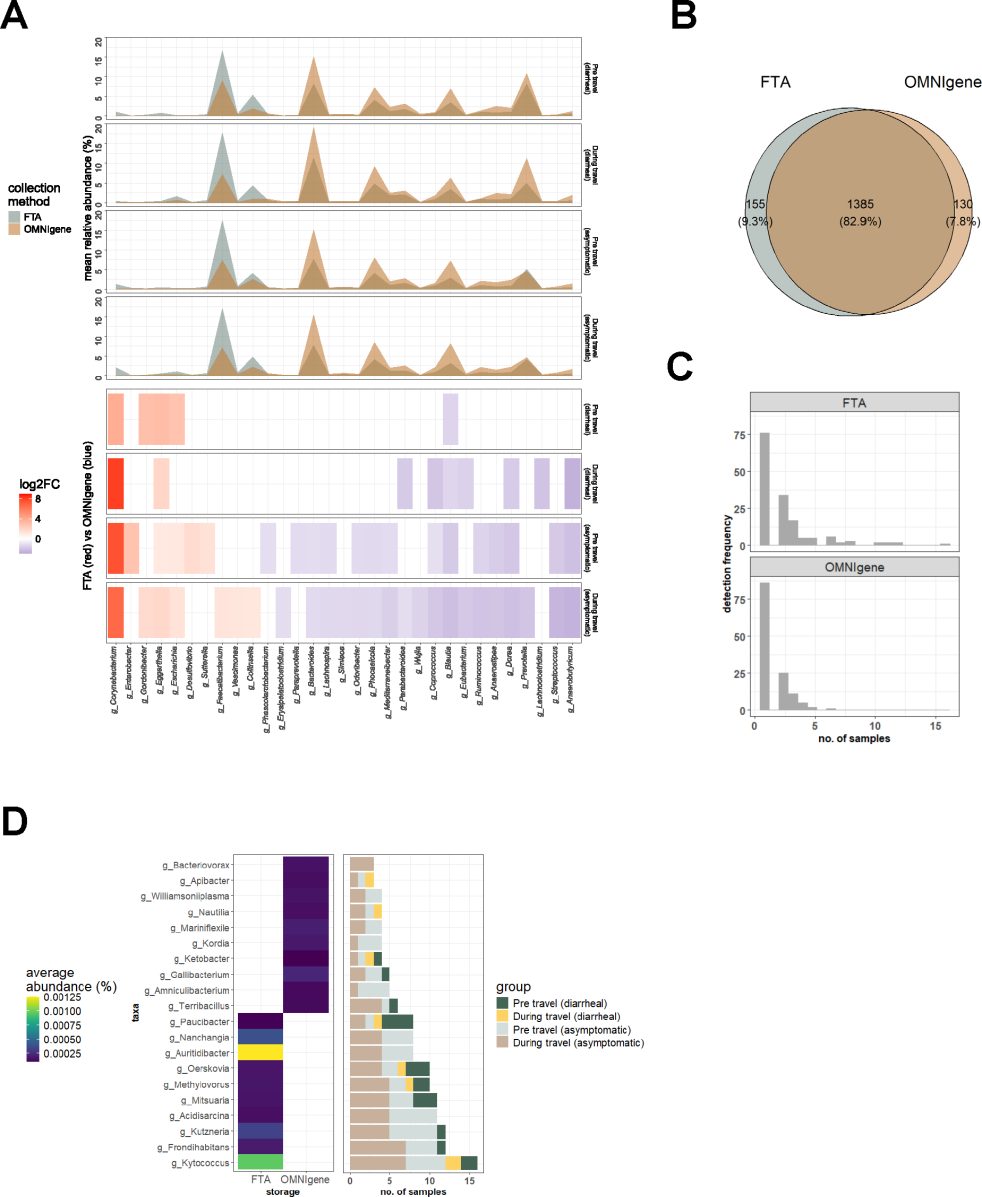
Taxonomic differences between FTA and OG samples. (**A**) Differential abundance analysis of genera between FTA and OG samples for each timepoint-disease group. The top panel shows the mean relative abundances of each genus per collection method (FTA; blue, OG; tan). The bottom panel shows the log2 fold change values of each genus for each timepoint-disease group (enriched in FTA; red, enriched in OG; blue). (**B**) The number and percent of genera that are shared and unique between FTA and OG samples. (**C**) The frequency of genera that is uniquely detected in FTA or OG samples. (**D**) Top 10 unique genera captured by FTA and OG samples, respectively. The left panel shows the mean relative abundance of each genus across samples per collection method. The right panel shows the prevalence of each genus across samples and is colored according to timepoint-disease groups.

Differential abundance analysis was used to identify genera that were significantly different (*P* < 0.05) between collection methods for each timepoint-disease state group (Fig. 2A). Genera that had a significantly higher abundance with FTA cards in at least two or more groups include *Corynebacter*, *Eggerthella*, *Escherichia,* and *Gordonibacter. Corynebacterium* had the highest magnitude of fold change and was found to be consistently higher in abundance across all four groups with FTA. A diverse range of *Corynebacterium* species, such as *C. simulans*, *C. kefirresidentii*, and *C. genitalium*, was observed. Conversely, genera that were significantly higher in OG in at least two or more groups include *Anaerobutyricium*, *Anaerostipes*, *Blautia*, *Coprococcus*, *Dorea*, *Eubacterium*, *Lachnospira*, *Mediterraneibacter*, *Odoribacter*, *Parabacteroides*, *Phocaeicola*, *Ruminococcus,* and *Streptococcus. Blautia* was observed to be consistently higher in OG across all four groups and includes species, such as *Blautia obeum* and *Blautia wexlerae*. Opposing shifts in differential taxa between collection methods were not observed across groups.

### Biases of collection methods towards specific taxa

Genera that were uniquely captured by each collection method were further investigated. The results indicate that 82.9% of genera were detected with both methods, while FTA and OG uniquely captured 9.3% and 7.8% of genera, respectively (Fig. 2B). These unique genera, however, are present in extremely low relative abundances (<0.01%) and are not highly prevalent across samples (Fig. 2C and D). FTA-specific genera include skin microbes, such as *Kytococcus* and *Auritidibacter*, and environmental species, such as *Kutzneria* and *Frondihabitans,* while genera that were uniquely captured by OG include environmental species such as *Nautilia* and *Ketobacter*.

## DISCUSSION

The collection of fecal samples in deployed military environments is challenging and impedes the ability to conduct field microbiome research in a consistent and reproducible manner. Thus, there is a need to evaluate field expedient collection methods that are easily deployable in real-world settings. Our analyses included comparisons at two different time points (pre- and during travel) and between subjects who remained asymptomatic or experienced travelers’ diarrhea—both variables that could impact microbiome composition (22–24) and subsequent capture of different species by each collection method.

While the recovery of nucleic acid and subsequent number of sequenced reads from FTA cards was significantly lower compared to OG kits, its impact on species richness is not apparent as indicated by similar Shannon diversity and the number of observed species. While no significant differences in Shannon diversity between OG and FTA were observed, the significantly higher Simpson diversity in OG samples compared to FTA suggests that microbial communities captured by OG are more evenly distributed among their dominant species. The lower recovery of nucleic acid from FTA cards has also been observed previously (14, 25) and is likely proportional to the amount of stool smeared onto the cards, suggesting that sampling with FTA cards is more reliant on proper usage compared to the OG kit.

Differences in microbial composition between the two collection methods are apparent as reflected by significant beta diversity differences and the identification of differentially abundant taxa along with the presence of unique species captured by each method. Interestingly, our analysis showed consistent enrichment of Actinobacteria species, including *Corynebacterium*, *Eggerthella,* and *Gordonibactor* in FTA samples across all timepoint-diarrhea groups, with these enrichment differences being consistent across distinct time points and health states. The enrichment of *Corynebacterium* in FTA samples is likely attributed to collection practices, whereby participants were advised to smear the FTA card with soiled toilet paper (see Materials and Methods). In addition, the presence of commensal skin microbes that were uniquely captured by FTA also suggests a higher probability of direct skin contact with the FTA card during sampling. On the other hand, a lower risk of such contamination was observed with the OG kit, likely resulting from the use of a spatula for sample transfer that is included in the kit. This observation recommends the use of a similar apparatus to minimize surrounding contamination during sample collection and handling. Furthermore, given that low biomass samples are more susceptible to contamination by exogenous microbes (26, 27), future studies are needed to evaluate the impact of varying biomass levels on microbiome recovery using FTA cards. Collectively, along with the higher recovery of nucleic acid, the OG kit appears to be a more user-friendly alternative for sample collection and preservation.

Similar to previous reports (28, 29), we observed that on average, relative abundances of Bacteroidetes were higher in OG samples compared to FTA. Differential abundance analyses at the genus level indicate that this was driven by specific Bacteroidetes genera (i.e., *Bacteroides, Odoribacter, Parabacteroides, Paraprevotella, Phocaeicola,* and *Prevotella*). We also observed a depletion of *Faecalibacterium* in OG samples, which is consistent with previous findings (29). Similarly, previous studies have reported either a higher abundance of Actinobacteria in FTA compared to frozen stool or a high concordance of Actinobacteria between FTA and frozen stool (12, 15, 25, 30). While the composition of chemicals impregnated in the FTA cards is unknown, this observation could be attributed to the efficient chemical lysis of Actinobacteria, which consists mostly of Gram-positive species with thick peptidoglycan layers.

While the collection method appeared to have a significant effect on community composition, the largest source of microbiome variation was explained by interindividual differences. This observation aligns with previous studies conducting similar assessments, whereby inter-individuality was found to have the largest impact on microbiome composition (12, 31, 32). Overall, while consistent use of a single collection method within a study is important to minimize confounding effects for downstream analysis, these observations indicate that variables of interest with a large effect size on the microbiome would be sufficiently captured regardless of collection method, particularly if a binary presence/absence metric is desired. Furthermore, genera that were uniquely captured by each method are shown here to be low abundant non-gastrointestinal species, and therefore such distinctions should not confound findings from fecal microbiome studies specifically. Nevertheless, the interpretation of low-abundant organisms is challenging due to the increased chances of false positives derived from sequencing errors and sequence contamination (33).

FTA cards have been shown to be plausible for long-term biobanking of samples that are up to a decade old (10, 34), while the stability of OG samples up to 18 months was also previously described (28). This could be important for samples derived from unique cohorts and environments that are difficult to replicate, such as the ones presented in this study. At ambient temperatures, the current manufacturer recommendation for OG kits is 60 days compared to indefinite storage using FTA cards. Aside from storage efficacy, cost is also an important determining factor for method selection, especially for large-scale studies. A previous 2021 assessment comparing the cost of several fecal storage methods suggests that FTA could be a cost-effective option at USD 7.52 per unit compared to OG kits at USD 20.00 per unit (11).

While the systematic collection of paired FTA and OG samples across different time points and health states allowed for comprehensive and direct comparisons between the two collection methods, our study poses several limitations. Due to technical constraints, it was not possible to collect freshly frozen fecal samples from deployed individuals for direct benchmarking of methods. Nevertheless, many prior studies have shown that FTA and OG are effective room-temperature storage alternatives when compared to frozen stool (8, 11, 13, 15). Also, our analysis was purely focused on metagenomics and did not consider methods that are suitable for metatranscriptomics, metaproteomics, and metabolomics analyses. In fact, previous reports have indicated that 95% ethanol appears to be the best storage method for metabolomics measurements from fecal samples (11, 15, 35). Future assessments would be required to investigate the feasibility of using field-compatible methods for other types of multi-omics applications. Moreover, the paired longitudinal samples collected as part of this study will allow for future analyses on the impact of travel and traveler’s diarrhea on the gut microbiome in a deployed setting.

### Conclusion

This study presents a systematic comparison of field sample collection methods for fecal metagenomics on a unique cohort of active-duty personnel in deployed environments. Our data were obtained from a military operational environment, thereby reflecting conditions that would be difficult or impossible to replicate in a laboratory setting. These results summarize global and specific distinctions between the two methods, which could serve as a reference for the design of future collection protocols for fecal metagenomics analysis across a wide range of applications. Overall, our findings indicate the importance of using consistent collection methods for comparisons within and across studies and the importance of understanding biases of fecal collection and storage protocols that could affect downstream conclusions.

## MATERIALS AND METHODS

### Study design and sample collection

We used stool samples collected as part of an ongoing, double-blind, placebo-controlled clinical trial evaluating the effectiveness of a food supplement for the maintenance of gut health during deployment and travel to regions with a risk of travelers’ diarrhea (36) for a duration of 10 days to 2 months. Supplement administration status was not available to the authors. However, both the FTA Elute Card and OMNIgene kit (OMR-200; DNA Genotek, Ontario, Canada) collection procedures were applied to all individuals from all study groups, thereby mitigating confounding effects on the direct collection method comparison assessed specifically in this study.

Active duty service members from the U.S. Department of Defense (DoD) and United Kingdom Ministry of Defence and adult U.S. DoD beneficiaries were enrolled in the trial after providing informed consent. U.S. participants self-collected a stool sample in an OG kit and a stool smear on an FTA card prior to travel and starting the study product and mailed the sample to a central laboratory for storage and extraction. Paired OG and FTA samples per participant were collected from the same stool sample between 10 and 20 days after arrival in the destination, and up to two additional FTA samples were collected if the participant experienced diarrhea. Participants followed the manufacturer’s instructions provided in the OG kit by using a spatula to insert a small amount of feces in the tube. Participants were instructed to smear FTA cards using soiled toilet paper after using the toilet. Gastrointestinal symptoms and any antibiotic or antimotility treatment were recorded in a travel diary. U.S. participants mailed the diary and samples to the central laboratory after return to the United States. Pre-travel and during-travel stool samples for UK participants were collected in stool hats and aliquoted by study staff into OMNIgene Gut Tubes and FTA elute cards and shipped to the central laboratory in the United States at room temperature. Upon receipt in the laboratory, FTA cards and OMNIgene tubes were refrigerated until extraction. For the current analysis, we included a subset of asymptomatic subjects who submitted paired (i.e., OG and FTA) pre-travel and paired during-travel samples. We also included subjects who reported travelers’ diarrhea (defined as ≥3 loose stools in a 24 h period or >2 loose stools plus abdominal symptoms (i.e., abdominal cramps, nausea, vomiting, or fever) in a 24 h period and collected an FTA card during a diarrheal episode in addition to the paired pre- and during travel stool samples.

Subjects included in this analysis were enrolled at four sites: Tripler Army Medical Center, Honolulu, HI; Naval Medical Center Portsmouth, Portsmouth, VA; Madigan Army Medical Center, Tacoma WA; and Research and Clinical Innovation, UK Ministry of Defence, UK.

### Nucleic acid extraction

DNA was extracted from fecal samples collected using FTA as described previously (18). Briefly, three 3 mm disc punches were excised from the sample area of the FTA card and washed by pulse vortexing with 500 μL of sterile water. The discs were subsequently transferred to 100 μL of sterile water, subjected to bead beating (acid-washed glass beads) at maximum speed for 2 min, and incubated at 95°C for 5 min. Eluted DNA was collected following 30 s centrifugation. DNA from OG tubes was extracted with the QIAamp Power Fecal DNA Kit (Qiagen) using 250 μL of sample according to manufacturer’s instructions. DNA was quantified across all samples using a Qubit fluorometer (Thermo Fisher) prior to library preparation and sequencing.

### Metagenomic sequencing and classification

Whole metagenomic sequence libraries were prepared from gDNA via the Illumina DNA Prep library preparation kit and sequenced on the Illumina NextSeq 2000 via P2 300 cycle reagents and flow cell. No-template negative controls and FTA and OG blanks were also included. Sequencing data were quality filtered using fastp (37), and reads were aligned to the human reference genome, GRCh38, for removal of host reads using minimap2 (38). Post-processed sequences were taxonomically classified via centrifuge (39) using a January 2023 custom build of the NCBI BLAST nucleotide reference database (40). Briefly, this comprehensive database includes sequences from all domains of life with quality control measures, such as cross-kingdom sequence decontamination and removal of sequences shorter than 16 nucleotides. The indexed database is available for public use at https://benlangmead.github.io/aws-indexes/centrifuge. Centrifuge profiles were re-processed using Recentrifuge (41), with a minimum hit length of 40, and taxonomic counts were generated for each sample.

### Microbiome analysis and statistics

Microbiome profiles were imported into phyloseq (42) for downstream diversity analyses. DESeq2 (43) was used to determine differential abundant taxa. PERMANOVA was carried out with the vegan package (44) to determine the significance of beta diversity (Bray-Curtis and Jaccard) and to estimate the percentage of variability explained (R2) by each variable. An ANOVA of mixed linear models was used to estimate the effect of each variable on the first 10 axes of the principal coordinate analysis (PCoA). Wilcoxon rank-sum tests were used for bivariate comparisons, followed by Bonferroni adjustments for pairwise comparisons and FDR adjustment for multiple comparisons.

## Data Availability

The raw data sets generated in this study are not publicly available due to privacy sensitivities regarding military service member cohorts, but deidentified aggregate data sets are available from the authors on reasonable request and in accordance with applicable regulations and data usage agreements.

## SUPPLEMENTAL MATERIAL

Supplemental figures msphere.00818-24-s0001.pdf Fig. S1 and S2.

Table S1 msphere.00818-24-s0002.csv Taxonomic counts.

ASM does not own the copyrights to Supplemental Material that may be linked to, or accessed through, an article. The authors have granted ASM a non-exclusive, world-wide license to publish the Supplemental Material files. Please contact the corresponding author directly for reuse.
