# Supplementary material for: Field expedient stool collection methods for gut microbiome analysis in deployed military environments
Source: mSphere. 2025 May 15;10(6):e00818-24. doi: 10.1128/msphere.00818-24 (PMC12188722; doi:10.1128/msphere.00818-24)
Supplement: Supplemental figures — Fig. S1 and S2. [file msphere.00818-24-s0001.pdf]

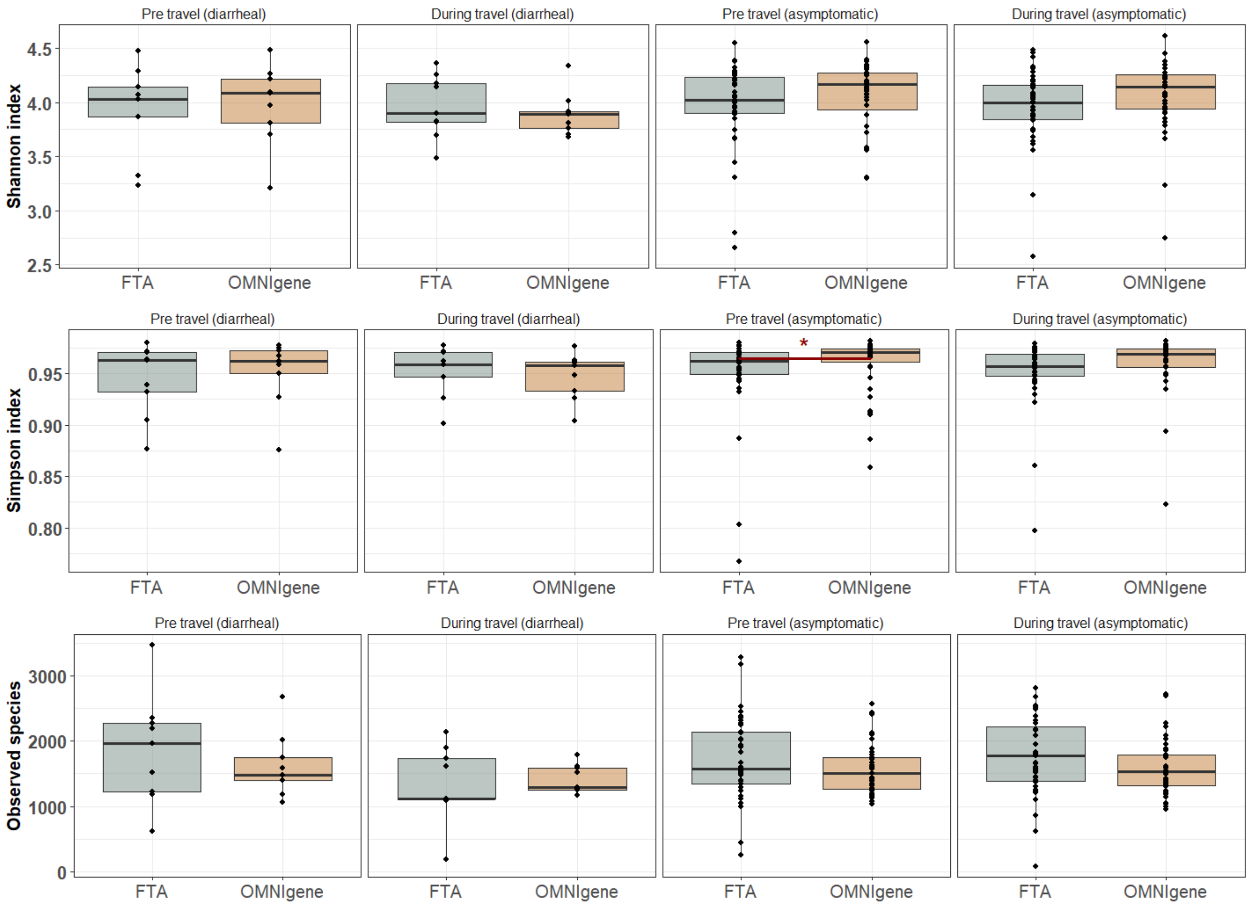

Fig S1. Alpha diversity measurements (The number of observed species, Shannon index and Simpson index) across samples grouped according timepoint-disease categories (*; p<0.05).

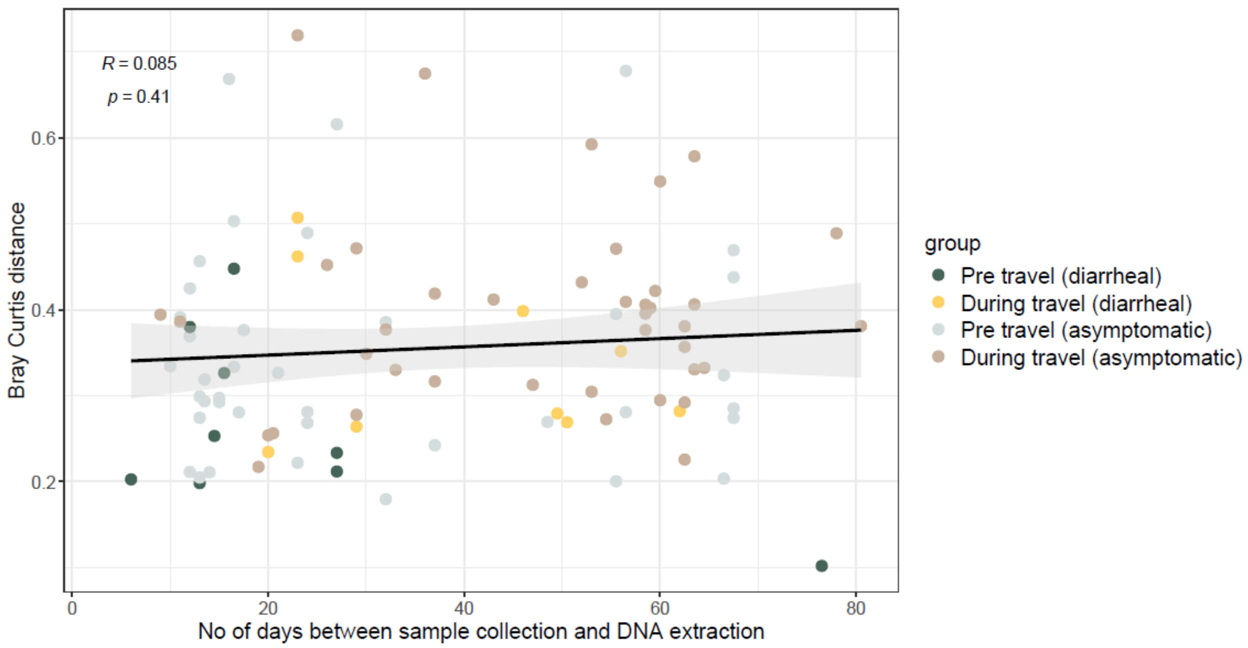

Fig S2. Pearson correlation between the number of days elapsed from sample collection to extraction and the Bray Curtis dissimilarity index for paired OG and FTA samples.
